# Waiting time for surgery and health-related quality of life in patients with inflammatory bowel disease

**DOI:** 10.1007/s00384-026-05258-9

**Published:** 2026-10-23

**Authors:** Hanna de la Croix, Helena Åberg, Stephanie Fledsberg, Jennifer Park, Jonas Bengtsson, Maria Hermanson

**Affiliations:** 1 Department of Surgery, IBD-SURG-Inflammatory Bowel Disease Surgical Research Group, Institute of Clinical Sciences, Sahlgrenska Academy, University of Gothenburg, Gothenburg, Sweden; 2 Department of Surgery, Sahlgrenska University Hospital, Region Västra Götaland, Gothenburg, Sweden; 3 Gothia Forum for Clinical Trials, Region Västra Götaland, Sahlgrenska University Hospital, Gothenburg, Sweden; 4 Department of Surgery, SSORG-Scandinavian Surgical Outcomes Research Group, Institute of Clinical Sciences, Sahlgrenska Academy, University of Gothenburg, Gothenburg, Sweden

**Keywords:** Inflammatory bowel disease, IBD surgery, Surgical waiting time, Health-related quality of life, Short Health Scale, Socioeconomic status, Registry-based study

## Abstract

**Purpose:**

When surgery is indicated in patients with inflammatory bowel disease (IBD), timely intervention may reduce the risk of further complications. This study aimed to assess health-related quality of life (HRQoL) in relation to waiting time for IBD surgery.

**Methods:**

This cross-sectional, register-based study assessed HRQoL using the EQ-5D-5L questionnaire. Patients with IBD who had undergone surgery or were awaiting surgery between 2017 and 2023 at Sahlgrenska University Hospital, Gothenburg, Sweden, were identified and invited to participate.

**Results:**

A total of 214 patients were included in the study (33.6% women, mean age 44.3 ± 16.9 years). Sixty percent had undergone surgery, while the remainder were awaiting surgery. The mean EQ VAS was 64.9, and the lowest scores were observed in patients waiting for surgery for over a year, compared to Swedish age-matched population norms, with a mean EQ VAS of 87.0. Using the Swedish value set, the mean EQ-5D-5L index was higher among patients waiting for surgery < 3 months compared with those waiting 12–24 months (*p* = 0.017) and > 24 months (*p* = 0.012). It was also higher among patients waiting 3–6 months compared with those waiting 12–24 months (*p* = 0.036) and > 24 months (*p* = 0.032).

**Conclusion:**

Prolonged waiting time for IBD surgery was associated with impaired HRQoL, particularly among patients who had waited more than 1 year. While causal inferences cannot be drawn from this cross-sectional study, the findings support incorporating patient-reported HRQoL into prioritisation of patients awaiting IBD surgery.

**Trial registration:**

The study has been registered in clinicaltrials.gov (NCT: 2022-05975-01).

**Supplementary Information:**

The online version contains supplementary material available at https://doi.org/10.1007/s00384-026-05258-9.

## Introduction

Compared with surgery for malignant conditions, surgical procedures for inflammatory bowel disease (IBD) have been subject to longer delays in many countries, with further postponements observed during the COVID-19 pandemic [1–5]. Activity in IBD is associated with an increased number of IBD-related surgical procedures, hospitalizations, and work productivity impairment, all of which impairs Health Related Quality of Life (HRQoL) [6, 7]. Further, IBD patients waiting for surgery often experience severe symptoms such as intestinal bowel obstruction, perforations, and fistulas. Others may experience intolerable side effects of medications [7, 8]. When surgery is delayed in IBD patients, health economics has been shown to be affected, including an increase in overall health care costs [9]. Also, longer waiting period for general surgery is marked by a decline in health and adverse effect on the patient’s mental well-being and social life [10]. In this context, socioeconomic status, geographic differences, and structural barriers may influence access to surgical care and contribute to further delays in timely treatment [11, 12].

Using registry data and HRQoL questionnaires, this study aims to examine the association between waiting time for surgery and HRQoL in patients with IBD at a tertiary referral centre. Specifically, it compares individuals awaiting surgery with those who received timely intervention within three months of surgical decision-making and assesses whether socioeconomic factors are associated with surgical waiting time.

## Methods

This cross-sectional, register-based study integrated individual-level data from patients with IBD who were either scheduled for or had undergone surgery at Sahlgrenska University Hospital, a tertiary referral centre in Gothenburg, Sweden, between January 2017 and December 2023. Patients were identified using the administrative surgery planning system *Orbit* that provided information on surgical decision date, patient age, diagnosis code, mode of admission (elective or acute), and date of surgery. No formal sample size calculation was performed; all eligible patients identified during the study period were invited to participate. Data on sex were obtained from VEGA, the regional healthcare database in Region Västra Götaland. Socioeconomic data, including income, educational level, and country of birth, were obtained from Statistics Sweden’s longitudinal database LISA, provided through The Swedish Social Insurance Agency. Patient-reported outcomes were collected using the EQ-5D-5L and Short Health Scale (SHS) questionnaires and linked with registry data from the Swedish Inflammatory Bowel Disease Registry (SWIBREG). [13–15] This study was reported in accordance with the Strengthening the Reporting of Observational Studies in Epidemiology (STROBE) guidelines [16].

Income was defined as equivalized disposable household income, including income from work and business activities, capital income, sickness, unemployment- or parental benefits, pension, child, housing- and study allowance minus final taxes. Income was adjusted for household composition using the consumption unit scale applied in Sweden's official statistics [17]. The most recent available income data were from 2022. If income data were unavailable for the current year, data from the latest available year were utilized.

The EQ-5D-5L is a validated generic instrument developed by the EuroQol Group to assess HRQoL across disease areas [13, 18] and is commonly used in health economic evaluations [19]. It includes five dimensions: mobility, self-care, usual activities, pain/discomfort, and anxiety/depression. Each dimension has five response levels ranging from no problems to extreme problems/unable to perform the activity. Responses generate a five-digit health profile describing the individual's health state.

EQ-5D-5L index values were calculated using two Swedish value sets. The first was based on the EuroQol Valuation Technology protocol for EQ-5D-5L, hereafter referred to as Swedish value set EQ-VT [20] [21]. The second was an experience-based value set derived from survey responses in which participants completed the EQ-5D-5L and valued their health using an open-ended time trade-off (TTO) question, hereafter referred to as Swedish value set experience-based [22]. The EQ-5D-5L also includes a visual analogue scale, the EQ VAS, on which patients rate their current health on a scale from 0 to 100. A score of 0 indicates the worst imaginable health state, while a score of 100 represents the best imaginable health state.

The Short Health Scale (SHS) is a validated four-part questionnaire designed to assess the impact of IBD on HRQoL [14, 23, 24]. The four dimensions in SHS include symptom burden, social function, disease-related worry, and general well-being. To reduce selection and non-response bias, eligible patients were identified systematically through the surgical planning register. The EQ-5D-5L/SHS questionnaires were distributed through mail and non-responders received two postal reminders and, when necessary, a telephone reminder. The entire data collection process, including distribution and reminders, spanned approximately nine months in total.

Patients were categorized into six groups according to waiting time for surgery: < 3 months, 3–6 months, 6–12 months, 12–24 months, > 24 months, and acute surgery defined as surgery performed within ≤ 14 days. Waiting-time categories were chosen to reflect clinically meaningful intervals in surgical prioritisation and follow-up. A waiting time of less than three months was considered to represent relatively timely surgery, in line with the Swedish healthcare guarantee for planned specialist care. Although patients may be referred to another healthcare provider if this guarantee cannot be fulfilled, this option is often limited for patients requiring complex IBD surgery, as such procedures are generally performed in specialised centres and are rarely available through private providers. Waiting times exceeding 12 months were considered prolonged. The categories were also selected to allow comparison between shorter, intermediate, and longer waiting times while retaining sufficient numbers of patients in each group.

Patients who underwent acute surgery were analysed as a separate group. Individuals who had acute surgery while waiting for elective surgery were categorized based on the waiting time either up to the acute surgery (if no elective surgery followed) or until the elective surgery (if it was performed following the acute surgery). For individuals with multiple surgeries, the latest recorded surgery decision date was used. The time between the surgery decision and the date of recorded EQ-5D-5L/SHS measure was estimated.

### Statistical analysis

The variables included in the analysis were demographic and clinical characteristics including age at surgical decision, sex, IBD-diagnosis, surgical decision date, surgery date, questionnaire completion date and waiting time for surgery. Socioeconomic variables included equivalized household income, country of birth, and educational level.

As this was a register-based study, the analyses were limited to variables systematically available for the full cohort. Detailed data on disease phenotype, disease activity, treatment exposure, nutritional status, disease duration, and surgical indication were unavailable and could therefore not be included as covariates.

The percentage of respondents reporting problems in different EQ-5D-5L dimensions, mean VAS scores, and mean EQ-5D-5L index were calculated for the various patient groups. A one-way ANOVA test was conducted to compare the EQ-5D-5L index values among different patient groups based on their waiting time for surgery. Differences were considered statistically significant at a *p*-value of < 0.05. Post hoc comparisons using the Tukey HSD test was then conducted. Analyses were based on available data for each variable, and no imputation of missing values was performed.

All analyses were performed using Stata version 17.0 (StataCorp, College Station, Texas, USA). Ethical approval was obtained by the Swedish Ethical Review Authority (Dnr 2022–05975-01) prior to data extraction. The study has been registered in clinicaltrials.gov (NCT: 2022–05975-01).

## Results

**Fig. 1:**
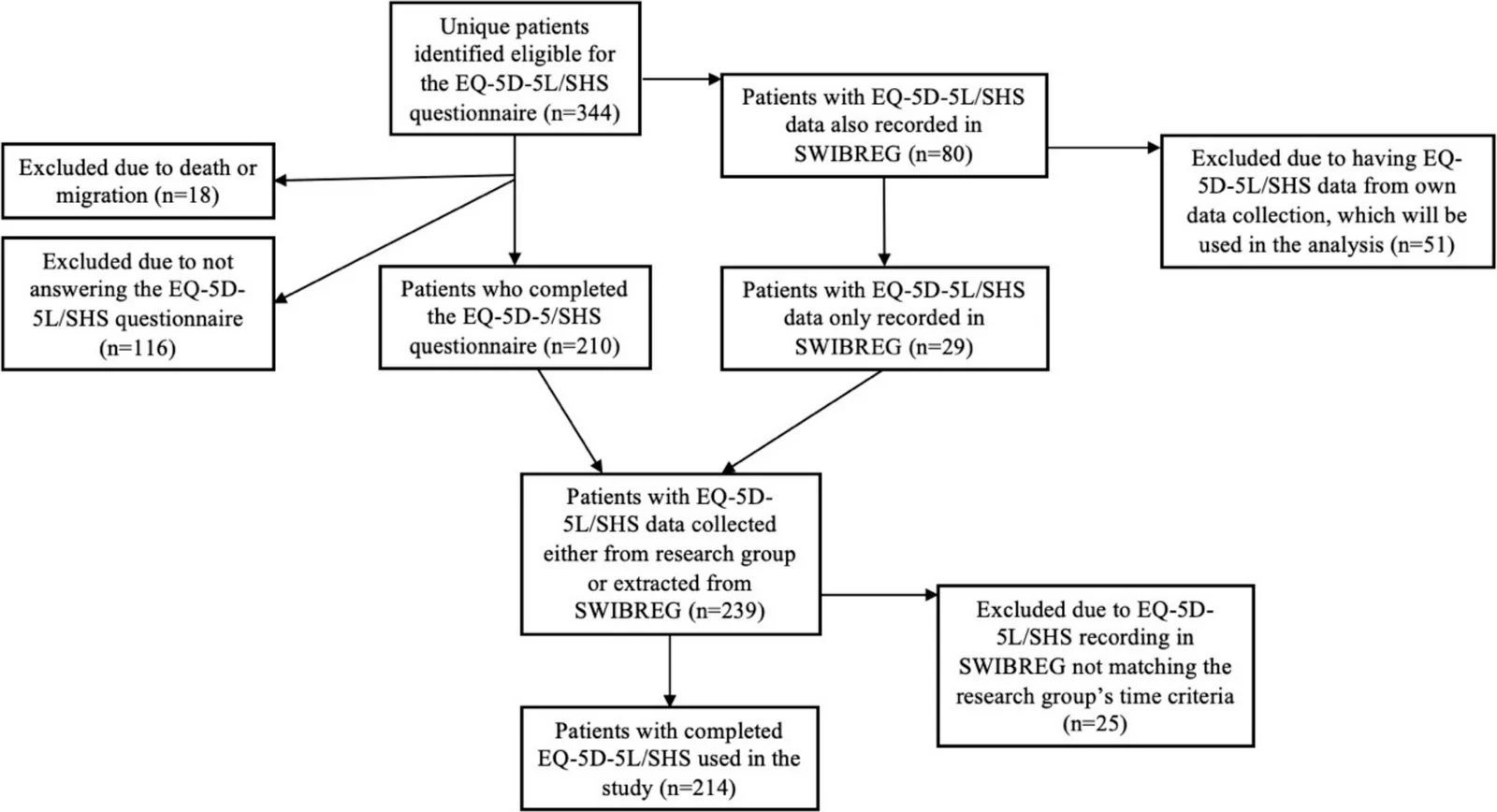
Flow chart of included patients with inflammatory bowel disease that had undergone surgery or were waiting for surgery between 2017 and 2023 at Sahlgrenska University Hospital, Gothenburg, Sweden

A total of 210 out of 344 individuals answered questionnaires by mail while data on EQ-5D-5L and SHS were supplemented for four individuals from the SWIBREG registry (62% response rate) (Fig. 1).

Background characteristics of the included individuals are shown in Table 1. Seventy-two were women (33.6%) and 142 men (66.4%) with a mean age at the time of surgical decision of 47 and 43 years, respectively. A total of 48 (22.4%) patients had Crohn’s disease, 162 (75.7%) had ulcerative colitis, and 4 (1.8%) had IBD-U (IBD unclassified). By the end of follow-up, 60% had undergone surgery whilst the remainder were still awaiting surgery. Finally, 65 individuals had waited longer than 24 months and this group also contained the widest range of waiting time (Appendix, figure 1).

**Table 1:** Background characteristics presented by the different patient categories according to waiting time in line for surgery. Data are presented as number (%) or mean ± SD

	Waited < 3m	Waited 3–6m	Waited 6–12m	Waited l2–24m	Waited > 24m	Acute < = 14d	Total
	*n*= 37 (17.3)	*n*= 29 (13.5)	*n* = 21 (9,8)	*n* = 26 (12.2)	*n* = 65 (30.4)	*n* = 36 (16.8)	*n* = 214 (l00.0)
Sex, *n*(%)							
Female	14 (37.8)	9 (31.0)	6 (28.6)	5 (19.2)	22 (33.8)	16 (44.4)	72 (33.6)
Type of IBD, *n* (%)							
Crohn’s disease	7 (18.9)	7 (24.1)	6 (28.6)	5 (19.2)	13 (20.0)	10 (27.8)	48 (22.4)
Ulcerative colitis	30 (81.1)	22 (75.9)	15(71.4)	19 (73.1)	52 (80.0)	24 (66.7)	162 (75.7)
IBD Unclassified	0 (0.0)	0 (0.0)	0 (0.0)	2 (7.7)	0 (0.0)	2 (5.6)	4 (1.9)
Age at surgical decision, mean ± SD	41.8 ± 17.4	46.5 ± 16.1	42.8 ± 11.8	42.5 ± 19.4	43.1 ± 16.1	49.2 ± 18.9	44.3 ± 16.9
Undergone surgery, *n* (%)							
Yes	37 (100.0)	28 (96.6)	12 (57.1)	6 (23.1)	6 (9.2)	36 (l00)	125 (58.4)
No	0 (0.0)	1 (3.4)	9 (42.9)	20 (76.9)	59 (90.8)	0 (0.0)	89 (41.6)
Waiting (days) for surgery, mean ± SD	46.1 ± 21.3	129.6 ± 26.6	275.7 ± 48.1	515.6 ± 115.4	1603.0 ± 564.9	2.3 ± 2.8	602.6 ± 748.2
Years between surgical decisions and completed EQ-5D-5L/SHS, mean ± SD	3.4 ± 1.5	3.3 ± 1.6	2.4 ± 2.1	1.9 ± 1.7	4.2 ± 1.6	2.1 ± 1.4	3.2 ± 1.8

Socioeconomic characteristics are shown in Table 2. A total of 173 (80.8%) patients were Sweden-born. Among patients who waited < 3 months for surgery, 89.2% were born in Sweden, compared with 76.9% among those who waited > 24 months. Education levels were similar across waiting-time groups, while mean household income tended to be higher among patients with shorter waiting times (Appendix, Table 1).

**Table 2:** Educational level, born in Sweden or outside, and household income presented by the different patient categories according to waiting time in line for surgery. Data are presented as percent (%)

	Waited < 3m	Waited 3–6m	Waited 6–12m	Waited 12–24m	Waited > 24m	Acute < = 14d	Total
	*n* = 37	*n* = 29	*n* = 21	*n* = 26	*n* = 65	*n* = 36	*n* = 214
Born in Sweden(%)							
Yes	89.2	79.3	71.4	84.0	76.9	83.3	80.8
No	10.8	20.7	28.6	16.0	23.1	16.7	19.2
Educational level (%)							
Low	70.3	55.2	57.1	65.4	60.9	72.2	63.8
Medium	29.7	24.1	23.8	26.9	31.3	19.4	26.8
High	0.0	20.7	19.0	7.7	7.8	8.3	9.4
Household income, divided in 5 quintiles (%)							
Lowest	5.4	27.6	9.5	7.7	29.2	27.8	20.1
Lower-Middle	18.9	20.7	38.1	19.2	18.5	13.9	20.1
Middle	21.6	13.8	14.3	23.1	23.1	19.4	20.1
Upper-Middle	27.0	17.2	19.0	30.8	9.2	27.8	20.1
Highest	27.0	20.7	19.0	19.2	20.0	11.1	19.6

Education level is operationalized as the highest obtained educational degree starting with lower-secondary education (compulsory nine years or less) upper-secondary education (three years or less), university (two years or higher). Household income is presented in quintiles, dividing the population into five equal parts based on equivalized household income (lowest to highest income quintile)

Education level is operationalized as the highest obtained educational degree starting with lower-secondary education (compulsory 9 years or less) upper-secondary education (3 years or less), university (2 years or higher). Household income is presented in quintiles, dividing the population into five equal parts based on equivalized household income (lowest to highest income quintile)

A health profile of 11,111 (perfect health) was reported by 30 individuals (14.0% of the total study population), where 9 individuals (24.3%) waited < 3 months for surgery (Table 3).

**Table 3:** EQ VAS score and the EQ-5D-5L descriptive system presented by the different patient categories according to waiting time in line for surgery

	Waited < 3 m	Waited 3–6 m	Waited 6–12 m	Waited 12–24 m	Waited > 24 m	Acute ≤ 14d
	*n* = 37	*n* = 29	*n* = 21	*n* = 26	*n* = 65	*n* = 36
EQ VAS, mean ± SD	72.7 ± 19.5	75.0 ± 16.0	65.3 ± 18.2	54.6 ± 28.8	64.4 ± 21.2	64.9 ± 20.6
Mobility						
No problems (%)	81.1	89.7	76.2	53.8	61.5	52.8
Slight problems (%)	10.8	3.4	9.5	23.1	12.3	27.8
Moderate problems (%)	8.1	6.9	14.3	15.4	15.4	13.9
Severe problems (%)	0.0	0.0	0.0	7.7	9.2	2.8
Unable (%)	0.0	0.0	0.0	0.0	1.5	2.8
Self-care						
No problems (%)	94.6	96.6	85.7	80.8	76.9	83.3
Slight problems (%)	5.4	3.4	14.3	19.2	10.8	11.1
Moderate problems (%)	0.0	0.0	0.0	0.0	12.3	2.8
Severe problems (%)	0.0	0.0	0.0	0.0	0.0	2.8
Unable (%)	0.0	0.0	0.0	0.0	0.0	0.0
Usual activities						
No problems (%)	54.1	58.6	52.4	42.3	40.0	38.9
Slight problems (%)	21.6	24.1	14.3	23.1	26.2	38.9
Moderate problems (%)	18.9	6.9	19.0	11.5	21.5	5.6
Severe problems (%)	5.4	3.4	9.5	15.4	10.8	13.9
Unable (%)	0.0	6.9	4.8	7.7	1.5	2.8
Pain/discomfort						
No (%)	29.7	24.1	19.0	19.2	23.1	33.3
Slight (%)	37.8	58.6	47.6	34.6	33.8	27.8
Moderate (%)	27.0	10.3	19.0	26.9	24.6	30.6
Severe (%)	5.4	3.4	14.3	19.2	15.4	5.6
Extreme (%)	0.0	3.4	0.0	0.0	3.1	2.8
Anxiety/depression						
Not (%)	51.4	48.3	33.3	19.2	26.2	33.3
Slightly (%)	32.4	34.5	28.6	38.5	38.5	27.8
Moderately (%)	13.5	3.4	33.3	23.1	23.1	27.8
Severely (%)	2.7	13.8	4.8	7.7	9.2	8.3
Extremely (%)	0.0	0.0	0.0	11.5	3.1	2.8

**Fig. 2:**
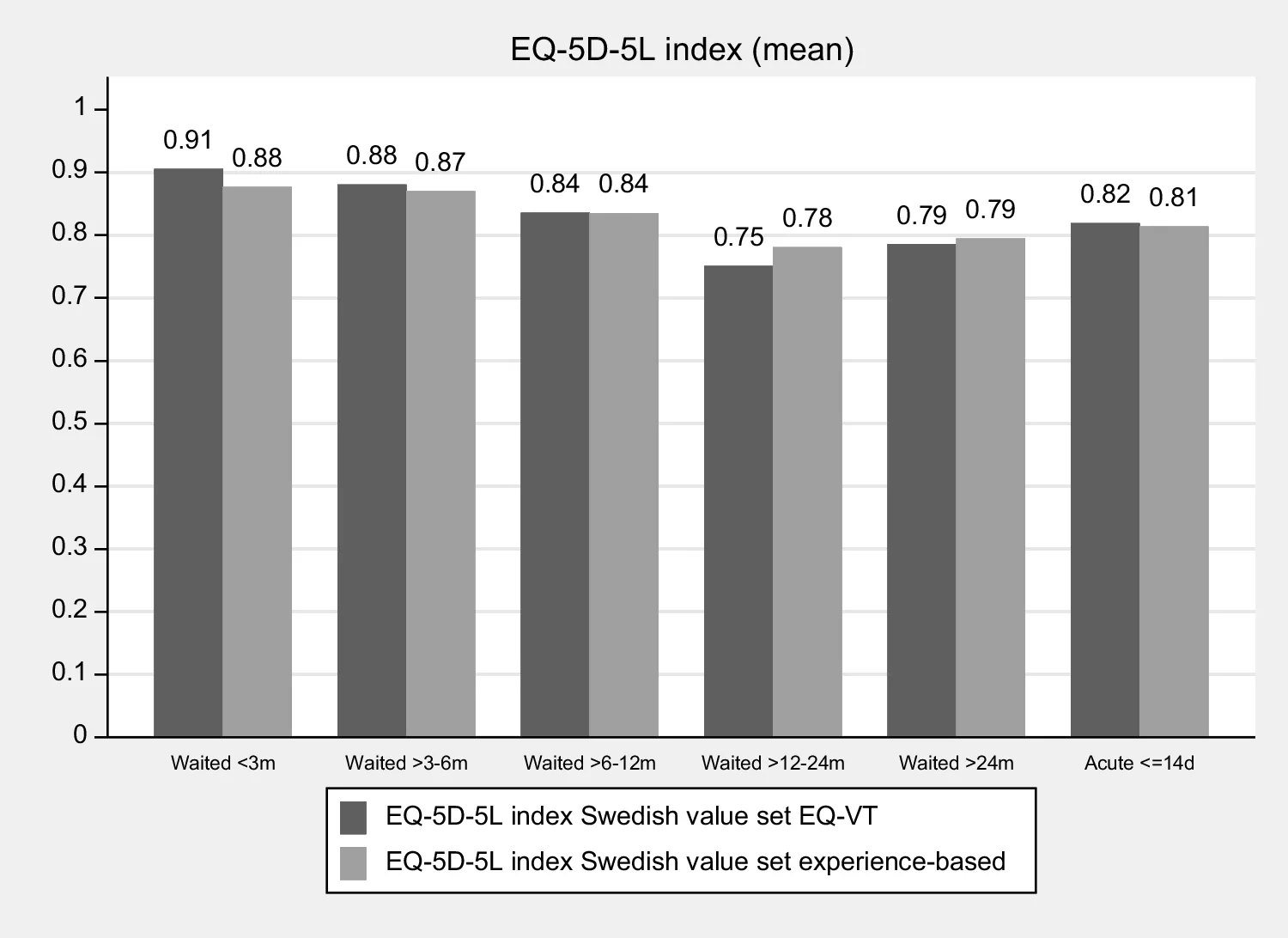
EQ-5D-5L index (mean) presented in a graph bar by patient category according to waiting time in line and calculated based on two different Swedish value sets

The lowest mean EQ-5D index was observed among those who had been waiting for surgery for more than a year but less than 2 years, with a mean index of 0.75 and 0.78, depending on used value set (Fig. 2). A separate analysis was conducted to assess the HRQoL by IBD type (appendix figure 2–3) and showed that patients with UC assessed their HRQoL higher. Mean EQ-5D-5L index values are presented by waiting-time category and IBD type in appendix (appendix, figure 4a-b).

The mean EQ-5D index for patients who waited < 3 months was significantly better than those who waited 12–24 months (*p*-value = 0.017) and those who waited > 24 months (*p*-value = 0.012) respectively. Likewise, the mean EQ-5D index for patients who waited 3–6 months, was significantly better compared to those who waited 12–24 months (*p*-value = 0.036) and those who waited > 24 months (*p*-value = 0.032), using the Swedish value set experience-based. Comparable outcomes were observed when using the Swedish EQ-VT value set.

A sensitivity analysis restricted to patients with ulcerative colitis showed a similar pattern. Lower EQ-5D index values were observed among patients who waited 12–24 months and more than 24 months (Appendix, sensitivity analysis 1–2).

**Fig. 3:**
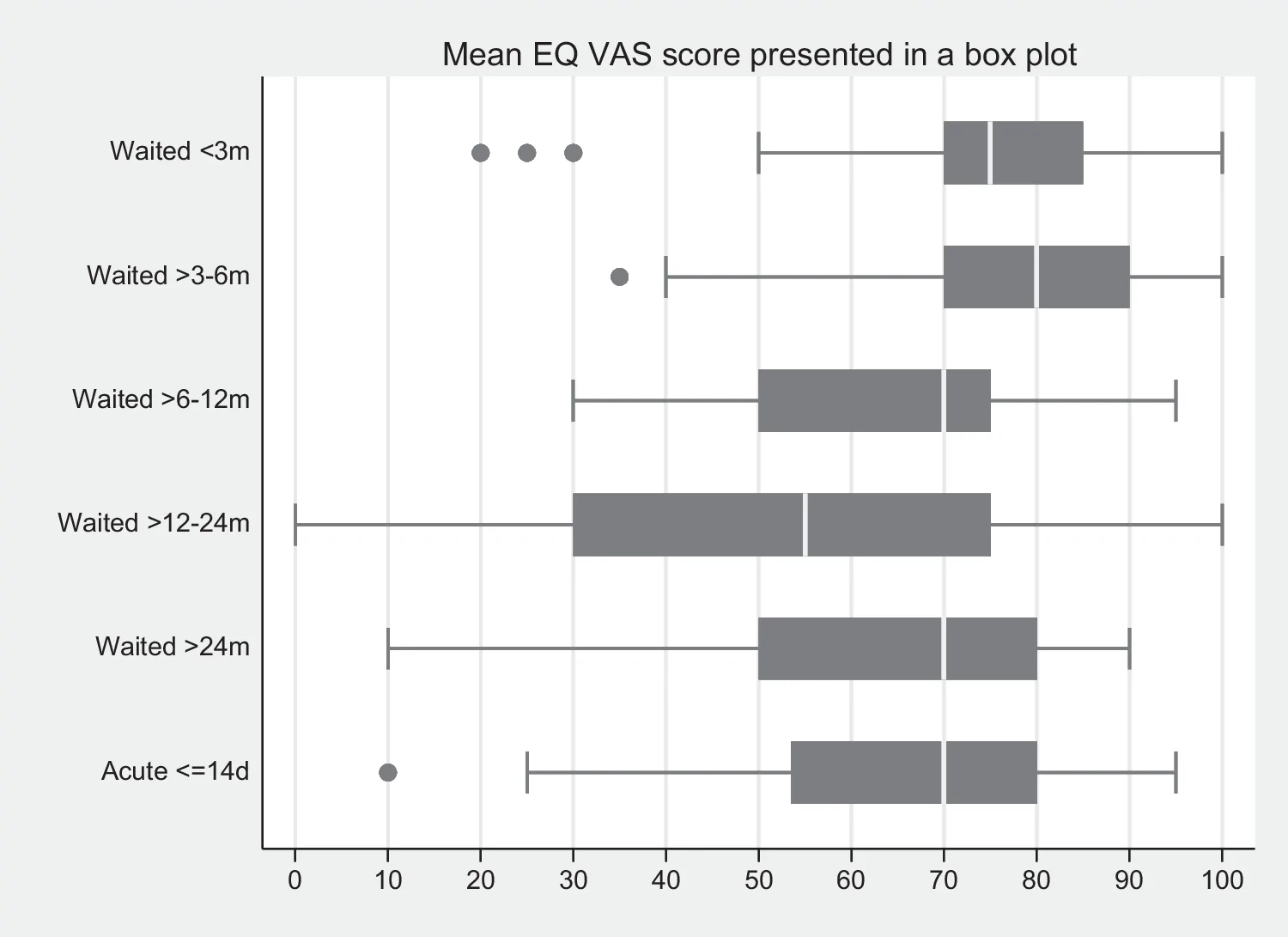
Distribution of mean EQ VAS score presented in a boxplot by patient category

The mean EQ VAS for the entire sample was 64.9 (Fig. 3), with the lowest scores observed among patients who had been waiting for surgery for more than 1 year but less than 2 years.

The analysis of SHS showed that longer waiting times for surgery were associated with greater worry and symptom severity, poorer general well-being, and more interference with daily life (Table 4), indicating that patients with longer waiting times reported higher proportions of severe outcomes. In patients who had waited 12–24 months, 15.4% answered that their IBD diagnosis interfered with daily life to a very high degree, compared to 0.0% among those who waited less than 3 months.

**Table 4:** Proportion of reported answers in SHS questionnaire assessing symptom severity, functional status, worry due to IBD, and the patient’s self-perception of their wellbeing

	Waited < 3 m	Waited 3–6 m	Waited 6–12 m	Waited 12–24 m	Waited > 24 m	Acute ≤ 14d
	*n* = 37	*n* = 29	*n* = 21	*n* = 26	*n* = 65	*n* = 36
Symptoms						
No symptoms (%)	27.0	20.7	4.8	26.9	13.8	27.8
Mild symptoms (%)	21.6	44.8	47.6	26.9	40.0	36.1
Moderate symptoms (%)	29.7	24.1	23.8	11.5	15.4	22.2
Rather severe symptoms (%)	16.2	6.9	19.0	15.4	21.5	13.9
Severe symptoms (%)	5.4	0.0	4.8	19.2	4.6	0.0
Very severe symptoms (%)	0.0	3.4	0.0	0.0	4.6	0.0
Interference in daily life						
Not at all (%)	24.3	27.6	19.0	15.4	7.7	13.9
To a low degree (%)	24.3	37.9	19.0	30.8	33.8	27.8
To a moderate degree (%)	24.3	20.7	28.6	19.2	21.5	38.9
To a fairly high degree (%)	13.5	3.4	14.3	11.5	20.0	11.1
To a high degree (%)	13.5	3.4	9.5	7.7	9.2	5.6
To a very high degree (%)	0.0	6.9	9.5	15.4	7.7	2.8
Worry due to IBD						
Not at all (%)	24.3	34.5	14.3	26.9	7.7	19.4
To a low degree (%)	24.3	34.5	23.8	11.5	27.7	27.8
To a moderate degree (%)	24.3	17.2	19.0	15.4	23.1	30.6
To a fairly high degree (%)	21.6	3.4	28.6	30.8	18.5	11.1
To a high degree (%)	5.4	3.4	14.3	11.5	16.9	8.3
To a very high degree (%)	0.0	6.9	0.0	3.8	6.2	2.8
General well-being						
Very good (%)	27.0	20.7	9.5	15.4	7.7	16.7
Good (%)	21.6	37.9	33.3	23.1	27.7	19.4
Fairly good (%)	43.2	31.0	33.3	30.8	38.5	41.7
Bad (%)	5.4	6.9	23.8	23.1	18.5	13.9
Very bad (%)	2.7	3.4	0.0	0.0	7.7	5.6
Terrible (%)	0.0	0.0	0.0	7.7	0.0	2.8

## Discussion

This cross-sectional, registry-based study found impaired HRQoL among patients with IBD who were scheduled for or had undergone surgery. Patients who had waited more than 1 year for surgery reported lower HRQoL than those who had undergone surgery within six months of the surgical decision. The lowest EQ-5D-5L index and EQ VAS scores were observed among patients waiting 12–24 months, suggesting that prolonged waiting time may be associated with a considerable burden of self-reported health.

Compared with previously reported Swedish population norms, HRQoL in the present study population was substantially lower. The mean EQ VAS score in this cohort was 64.9, compared with a reported mean of 87.0 among individuals aged 40–49 years in the general Swedish population [22]. Similarly, the EQ-5D index values observed in this study, particularly among patients waiting more than 1 year, were lower than previously reported Swedish population norms. These findings are consistent with previous studies showing that patients with IBD requiring surgery often have impaired HRQoL. Earlier research has also suggested that HRQoL may improve after surgery and, in some cases, approach levels observed in the general population during long-term follow up [25].

The association between longer waiting time and poorer HRQoL may reflect several mechanisms. Patients awaiting IBD surgery may experience persistent symptoms, complications, medication-related side effects, anxiety, and limitations in daily activities. The SHS results are consistent with this interpretation, as longer waiting times were associated with greater symptom burden, more disease-related worry, poorer general well-being, and greater interference with daily life. These findings are also in line with previous studies reporting complications among patients awaiting IBD surgery. Wasmann et al. reported that one in eight patients awaiting IBD surgery required emergent or semi-emergent surgery while on the waiting list [5].

However, the observed pattern was not strictly linear across waiting-time groups. Patients waiting more than 24 months did not report the lowest HRQoL, which could reflect patient selection, whereby patients with less severe or less urgent disease remained on the waiting list longer, while those with more severe disease were prioritised for earlier surgery. Differences in IBD subtype may also have contributed to this pattern. In the present study, a larger proportion of patients waiting more than 24 months had ulcerative colitis compared with those waiting 12–24 months, and previous studies have reported higher HRQoL among patients with ulcerative colitis than among those with Crohn’s disease [26].

A sensitivity analysis restricted to patients with ulcerative colitis showed a broadly similar pattern, with lower EQ-5D index values among patients with longer waiting times, particularly among those waiting more than 24 months. However, statistical significance was less consistent than in the full cohort, likely reflecting the smaller sample size. These findings suggest that the observed association between longer waiting time and impaired HRQoL was not solely driven by patients with Crohn’s disease, although the study was not powered to draw firm conclusions regarding diagnosis-specific associations.

Socioeconomic factors may play a role in access to surgical care. In the present study, mean household income tended to be higher among patients with shorter waiting times, and a larger proportion of patients waiting less than three months were born in Sweden compared with those waiting more than 24 months. These findings should be interpreted cautiously, as the socioeconomic analyses were descriptive and the study was not designed to establish causal relationships. Lower socioeconomic status is also independently associated with poorer self-reported health in many settings, and the present study cannot determine whether the observed differences reflect differences in access to surgery, underlying health disparities, disease-related factors, or other unmeasured confounding. Nevertheless, they are consistent with previous Swedish register-based research showing socioeconomic differences in surgical treatment among patients with IBD. Nordenvall et al. reported that restorative surgery after colectomy was more common among patients with higher income and less common among patients born outside the Nordic countries [27]. Taken together, these findings suggest that socioeconomic factors may be associated with surgical pathways, but this should be explored further in studies designed to assess equity in access to IBD surgery.

### Strengths and limitations

The main strength of this study is the combination of patient-reported HRQoL data with regional and national register data. The use of validated instruments, including the EQ-5D-5L and SHS, enabled assessment of both generic HRQoL and IBD-specific health burden. Furthermore, the study addresses an area that has been insufficiently explored in Sweden: the association between waiting time for IBD surgery and patient-reported outcomes.

This study also has several limitations. The cross-sectional and observational design precludes conclusions about causality. Patients were not randomized to different waiting times, and waiting time was likely influenced by clinical prioritization. Patients with more severe or urgent disease may have undergone surgery earlier, whereas patients with less urgent indications may have waited longer. Furthermore, because this was a register-based study, the analyses were limited to variables systematically available for the full cohort. Information on disease phenotype, clinical and endoscopic disease activity, inflammatory biomarkers such as C-reactive protein and faecal calprotectin, corticosteroid use, biologic or JAK inhibitor therapy, nutritional status, and disease duration was not consistently available. These factors may influence both waiting time and HRQoL and could therefore represent important sources of residual and unmeasured confounding. Consequently, the independent effect of waiting time on HRQoL cannot be determined from the present cross-sectional study.

Second, the timing of questionnaire completion varied, and HRQoL was measured at one point in time rather than longitudinally. Therefore, the reported health status may have been influenced by whether the patient was still awaiting surgery, had already undergone surgery, or had experienced postoperative recovery or complications. This was however, a deliberate design choice, as the study aimed to examine associations between waiting time from surgical decision-making and HRQoL in the overall surgical IBD cohort. Clinically, prolonged waiting may be relevant both before and after surgery, as patients may experience symptom burden, complications, and recovery-related difficulties. However, as HRQoL was measured at a single time point, we could not distinguish the potential impact of waiting time from postoperative recovery, adaptation, complications, pouch-related symptoms, stoma-related adjustment, or time elapsed since surgery. The results should therefore be interpreted as associations in a mixed pre- and postoperative cohort, rather than as evidence of a causal effect of waiting time.

Third, the limited sample size restricted the possibility of stratified analyses by IBD subtype, surgical indication, or type of procedure. Although a sensitivity analysis restricted to patients with ulcerative colitis showed a broadly similar pattern, the reduced sample size limited the possibility of drawing firm conclusions regarding diagnosis-specific associations. Differences between Crohn’s disease and ulcerative colitis in disease phenotype, surgical indication, type of procedure, and postoperative recovery may therefore still have influenced the observed associations.

Finally, although the response rate of 62% was achieved after several reminders and considered to be acceptable, non-response bias cannot be excluded. We know from previous studies that patients with the least problem show the least interest in answering questionnaires [28]. Hence, the HRQoL in this study-cohort could be underestimated.

Despite these limitations, the register-based design enabled systematic identification of patients referred for or undergoing IBD surgery over several years and allowed linkage with patient-reported HRQoL data collected using validated instruments. This provides real-world insight into HRQoL in a clinically relevant patient group that is difficult to capture systematically in routine clinical practice. However, as this was a single-centre study conducted at a tertiary referral centre, the generalisability of the findings to other healthcare settings may be limited. Taken together, the findings suggest that prolonged waiting time for IBD surgery may be associated with impaired HRQoL, particularly among patients waiting more than 1 year.

## Conclusion

This study found that prolonged waiting time for IBD surgery was associated with impaired HRQoL, particularly among patients waiting more than 1 year. Although causality cannot be inferred, the findings highlight the importance of considering patient-reported HRQoL in surgical prioritisation. Future prospective studies with repeated HRQoL assessments are needed to further evaluate the association between waiting time and HRQoL before and after IBD surgery.

## Supplementary Information

Below is the link to the electronic supplementary material.

ESM 1 (DOCX 55.4 KB)

## Data Availability

The administrative data used in this study are available from the National Board of Health and Welfare, the Swedish Social Insurance Agency, Statistics Sweden, and Region Västra Götaland. However, due to licensing and approval restrictions, we cannot make these data publicly available as it would violate the terms of use. Nevertheless, we are happy to provide information about the process of obtaining the data, as well as the code used for merging the data sets and conducting the analyses.
