# Supplementary material for: Waiting time for surgery and health-related quality of life in patients with inflammatory bowel disease
Source: Int J Colorectal Dis. 2026 Oct 23;41(1):170. doi: 10.1007/s00384-026-05258-9 (PMC13694504; doi:10.1007/s00384-026-05258-9)
Supplement: Supplementary file 1 [file 384_2026_5258_MOESM1_ESM.docx]

APPENDIX

**Appendix Figure 1.** Waiting time for surgery in months.

**Appendix Figure 2.** EQ-5D index (mean) by IBD type.

**Appendix Figure 3.** Mean EQ VAS by IBD type.

**Appendix Table 1.** Household income presented by patient groups in euros (average annual exchange rate for 2023: SEK 1 = EUR 0.08712).

| Patient group | Mean | Standard Error (SE) | 95% Confidence Interval |
| --- | --- | --- | --- |
| Waited <3 months | 39 667 | 4 852 | 30102-49232 |
| Waited 3-6 months | 29 161 | 3 016 | 23216-35105 |
| Waited 6-12 months | 27 759 | 2 300 | 23226-32292 |
| Waited 12-24 months | 30 572 | 1 773 | 27077-34068 |
| Waited >24 months | 28 634 | 2 436 | 23832-33435 |
| Acute <=14 days | 36 389 | 10 248 | 16189-56589 |

**Figure 4a.** EQ-5D-5L index (mean) presented in a graph bar by patient category according to waiting time in line and Crohn´s disease/Ulcerative colitis

Calculated using the EQ-5D-5L index Swedish value set EQ-VT (Sun Sun et al 2022)

**Figure 4b.** EQ-5D-5L index (mean) presented in a graph bar by patient category according to waiting time in line and Crohn´s disease/Ulcerative colitis

Calculated using the EQ-5D-5L index Swedish value set experience-based (Burström et al 2020)

**Figure 5a.** EQ-5D-5L index (mean) presented in a graph bar by patient category according to waiting time in line and surgery/no surgery.

Calculated using the EQ-5D-5L index Swedish value set EQ-VT (Sun Sun et al 2022)

**Figure 5b.** EQ-5D-5L index (mean) presented in a graph bar by patient category according to waiting time in line and surgery/no surgery.

Calculated using the EQ-5D-5L index Swedish value set experience-based (Burström et al 2020)

**Sensitivity analysis 1.** One-way ANOVA and post hoc pairwise comparisons of mean EQ-5D-5L index values across waiting-time categories, excluding patients with CD/IBD-U, using the Swedish experience-based EQ-5D-5L value set (Burström et al., 2020).

Although the overall ANOVA was not statistically significant (p = 0.204), a post-hoc comparison suggested that patients waiting >24 months had lower EQ-5D scores than those waiting <3 months (mean difference = −0.068, p = 0.047).

| Group Comparison | Mean Difference | Standard Error | t-value | p-value | 95% Confidence Interval |
| --- | --- | --- | --- | --- | --- |
| Waited >3–6m vs Waited <3m | -0.0029 | 0.0415 | -0.07 | 0.944 | [-0.0849, 0.0790] |
| Waited >6–12m vs Waited <3m | -0.0087 | 0.0467 | -0.19 | 0.853 | [-0.1010, 0.0837] |
| Waited >12–24m vs Waited <3m | -0.0775 | 0.0433 | -1.79 | 0.076 | [-0.1631, 0.0081] |
| Waited >24m vs Waited <3m | -0.0678 | 0.0339 | -2.00 | 0.047 | [-0.1347, -0.0008] |
| Acute ≤14d vs Waited <3m | -0.0304 | 0.0405 | -0.75 | 0.453 | [-0.1104, 0.0495] |
| Waited >6–12m vs Waited >3–6m | -0.0057 | 0.0495 | -0.12 | 0.908 | [-0.1035, 0.0920] |
| Waited >12–24m vs Waited >3–6m | -0.0746 | 0.0463 | -1.61 | 0.109 | [-0.1660, 0.0168] |
| Waited >24m vs Waited >3–6m | -0.0648 | 0.0376 | -1.72 | 0.087 | [-0.1391, 0.0094] |
| Acute ≤14d vs Waited >3–6m | -0.0275 | 0.0436 | -0.63 | 0.530 | [-0.1137, 0.0587] |
| Waited >12–24m vs Waited >6–12m | -0.0689 | 0.0511 | -1.35 | 0.179 | [-0.1697, 0.0320] |
| Waited >24m vs Waited >6–12m | -0.0591 | 0.0433 | -1.36 | 0.174 | [-0.1447, 0.0265] |
| Acute ≤14d vs Waited >6–12m | -0.0218 | 0.0486 | -0.45 | 0.655 | [-0.1179, 0.0743] |
| Waited >24m vs Waited >12–24m | 0.0098 | 0.0396 | 0.25 | 0.806 | [-0.0685, 0.0880] |
| Acute ≤14d vs Waited >12–24m | 0.0471 | 0.0454 | 1.04 | 0.301 | [-0.0426, 0.1368] |
| Acute ≤14d vs Waited >24m | 0.0373 | 0.0365 | 1.02 | 0.308 | [-0.0347, 0.1094] |

**Sensitivity analysis 2.** Same as above but calculated using the EQ-5D-5L index Swedish value set EQ-VT (Sun Sun et al 2022). Altough the overall ANOVA was not statistically significant (p = 0.153), the results indicate that participants who waited 12–24 months and more than 24 months had lower EQ-5D scores compared with those who waited less than 3 months (p = 0.033 and 0.029).

| Group Comparison | Mean Difference | Standard Error | t-value | p-value | 95% Confidence Interval |
| --- | --- | --- | --- | --- | --- |
| Waited >3–6m vs Waited <3m | -0.0294 | 0.0619 | -0.48 | 0.635 | [-0.1516, 0.0928] |
| Waited >6–12m vs Waited <3m | -0.0377 | 0.0697 | -0.54 | 0.589 | [-0.1754, 0.1000] |
| Waited >12–24m vs Waited <3m | -0.1393 | 0.0646 | -2.15 | 0.033 | [-0.2669, -0.0116] |
| Waited >24m vs Waited <3m | -0.1116 | 0.0505 | -2.21 | 0.029 | [-0.2115, -0.0118] |
| Acute ≤14d vs Waited <3m | -0.0408 | 0.0604 | -0.68 | 0.501 | [-0.1600, 0.0785] |
| Waited >6–12m vs Waited >3–6m | -0.0083 | 0.0738 | -0.11 | 0.910 | [-0.1541, 0.1375] |
| Waited >12–24m vs Waited >3–6m | -0.1098 | 0.0690 | -1.59 | 0.114 | [-0.2462, 0.0265] |
| Waited >24m vs Waited >3–6m | -0.0822 | 0.0561 | -1.47 | 0.145 | [-0.1929, 0.0285] |
| Acute ≤14d vs Waited >3–6m | -0.0113 | 0.0651 | -0.17 | 0.862 | [-0.1399, 0.1172] |
| Waited >12–24m vs Waited >6–12m | -0.1015 | 0.0761 | -1.33 | 0.184 | [-0.2519, 0.0489] |
| Waited >24m vs Waited >6–12m | -0.0739 | 0.0646 | -1.14 | 0.255 | [-0.2015, 0.0537] |
| Acute ≤14d vs Waited >6–12m | -0.0030 | 0.0726 | -0.04 | 0.967 | [-0.1463, 0.1403] |
| Waited >24m vs Waited >12–24m | 0.0276 | 0.0591 | 0.47 | 0.641 | [-0.0891, 0.1444] |
| Acute ≤14d vs Waited >12–24m | 0.0985 | 0.0677 | 1.46 | 0.148 | [-0.0352, 0.2322] |
| Acute ≤14d vs Waited >24m | 0.0709 | 0.0544 | 1.30 | 0.195 | [-0.0366, 0.1783] |
